# Analysis of pain markers and epidural fibrosis caused by repeated spinal surgery in Sprague–Dawley rats

**DOI:** 10.1186/s12891-020-03920-z

**Published:** 2021-01-05

**Authors:** Meiling Quan, Won-Ha Hwang, Jae-Hoon Kim, Young-Yul Kim

**Affiliations:** 1 Department of Pathophysiology, School of Basic Medical Sciences, Beihua University, Jilin, 132021 China; 2 Department of Orthopedics, Daejeon St. Mary’s Hospital, College of Medicine, The Catholic University of Korea, 64, Daeheung-ro, Jung-gu, Daejeon, 34943 Republic of Korea

**Keywords:** Fibrosis, Mitogen-activated protein kinases, Pain, Laminectomy

## Abstract

**Background:**

Epidural fibrosis is one of the aetiologies of pain following a spinal revision surgery. It is reported that the specific members of the mitogen – activated protein kinases (MAPK) family might mediate neuropathic pain. However, roles of epidural fibrosis caused by repeated spinal surgeries and pain-related proteins in causing the post spinal surgery syndrome remain unknown. Using a rat spinal surgery epidural fibrosis and adhesion model, in this study, we evaluated and investigated the relationship between pain markers and epidural fibrosis.

**Methods:**

Sprague–Dawley rats that underwent the spinal surgery were divided into three groups: group A (single laminectomy), group B (two repeated surgeries) and group C (three repeated surgeries). Dural thickness was measured in each experimental group, and immunohistochemical analysis and western blotting of mitogen-activated protein kinases were performed (ERK, p38 and JNK) using the spine cord.

**Results:**

Dural thickness was 6.363 ± 1.911 μm in group A, 13.238 ± 2.123 μm in group B and 19.4 ± 2.115 μm in group C, respectively. In the western blotting, phosphorylated ERK expression gradually increased with the number of repeated surgeries, and expression in groups B (1.77-fold) and C (2.42-fold) increased as compared to expression in group A. Phosphorylated p38 showed an increasing trend with the number of repeated surgeries, and groups B (1.17-fold) and C (1.33-fold) expression increased compared with group A. However, phosphorylated JNK expression did not gradually increase with the number of repeated surgeries, and groups B (1.62-fold) and C (1.43-fold) expression increased compared with group A. Excluding phosphorylated JNK, immunohistochemical analysis revealed that phosphorylated ERK and p38 expression gradually increased with the number of repeated surgeries in the spine dorsal horn, as evidenced by western blotting.

**Conclusions:**

Repeated spinal surgeries may increase dural thickness and expression of phosphorylated ERK and p38 in the spinal dorsal horn, and it suggests that the neuropathic pain is likely induced by epidural fibrosis and that the pain increases with the number of repeated surgeries.

**Supplementary Information:**

The online version contains supplementary material available at 10.1186/s12891-020-03920-z.

## Background

Each year, one million people worldwide undergo lumbar disc surgeries for disc herniation and spinal stenosis, making it one of the most common treatments for spinal diseases [1–4]. Despite appropriate decompression, however, the outcomes of such spinal surgeries are not necessarily correlated with the clinical outcomes. This may be because of the development of epidural fibrosis and adhesions, which is considered to be a normal reaction during healing following a spinal surgery [5]. It is well accepted that epidural fibrosis causes pulling, stretching or compression of the associated nerve root or dura mater and can lead to persistent back and leg pain following lumbar laminectomy, known as the post laminectomy syndrome, failed back surgery syndrome (FBSS) or post spinal surgery syndrome [6, 7]. Revision surgery in these cases has greater complication rates along with decreased success rate to 30% after the second, 15% after the third, and 5% after the fourth surgery [8]. In addition, such repeated surgeries may lead to dural tears, nerve root injuries and excessive bleeding [9]. Being one of the major constituents of post laminectomy syndrome or FBSS, epidural fibrosis is inevitable following a spinal surgery, and despite advancements in surgical techniques, some patients continue to suffer from recurrent postoperative pain [1]. It is difficult to expect good outcomes even when repeated surgeries are required to be performed to eliminate epidural fibrosis and adhesions.

Neuronal injury and post-injury regeneration progress as neural peptides and signal transduction molecules are expressed in the dorsal root ganglion (DRG) and spinal dorsal horn. In this regard, mitogen-activated protein kinases (MAPKs) are attracting much attention [10, 11]. MAPKs are serine/threonine protein kinases that conventionally comprise extracellular signal-regulated kinases 1/2 (ERK1/2), p38 and c-Jun N-terminal (JNK), which represent three different signalling pathway [12]. And MAPKs are activated by diverse extracellular stimuli, such as hormones and growth factors, and they transduce extracellular stimuli to intracellular transcriptional and post-transcriptional responses [10, 13]. Moreover, MAPKs are active participant in neuropathic pain and plasticity. For instance, if MAPKs are activated in the damaged neurons, and their inhibition can suppress of reverse neuropathic pain [14]. Increasing evidence has also shown that MAPKs play important roles in the induction and maintenance of chronic pain [15–17]. However, the roles of epidural fibrosis caused by repeated spinal surgery and pain-related proteins expressed in the spinal dorsal horn in causing the post spinal pain syndrome remain unknown.

In the present study, we evaluated the extent of epidural fibrosis by measuring dural thickness and investigated the association of MAPK expression in the spinal dorsal horn to study the relationship between epidural fibrosis and pain markers.

## Methods

### Animals

A total of 45 male Sprague–Dawley rats (age, 8 weeks; ORIENT BIO Inc., Korea) were used in this study. All animal experiments were approved by the Institutional Review Board of St. Mary’s Hospital of Catholic University (CMCDJ-AP-2012-017). The animals were randomly divided into three groups. In group A (*n* = 15), the rats underwent a single spinal laminectomy; in group B (n = 15), the rats underwent two laminectomies repeated at an interval of 3 weeks; and in group C (n = 15) the rats underwent three laminectomies repeated at intervals of 3 and 6 weeks (Table 1).

**Table 1:** Explanation regarding the progress of each experimental group

Group	Operation time	Operation data	Total survival
A	1		3 weeks
B	2	One more trial after 3 weeks	6 weeks
C	3	One more trial after 3 + 3 weeks	9 weeks

### Surgery

**Fig. 1:**
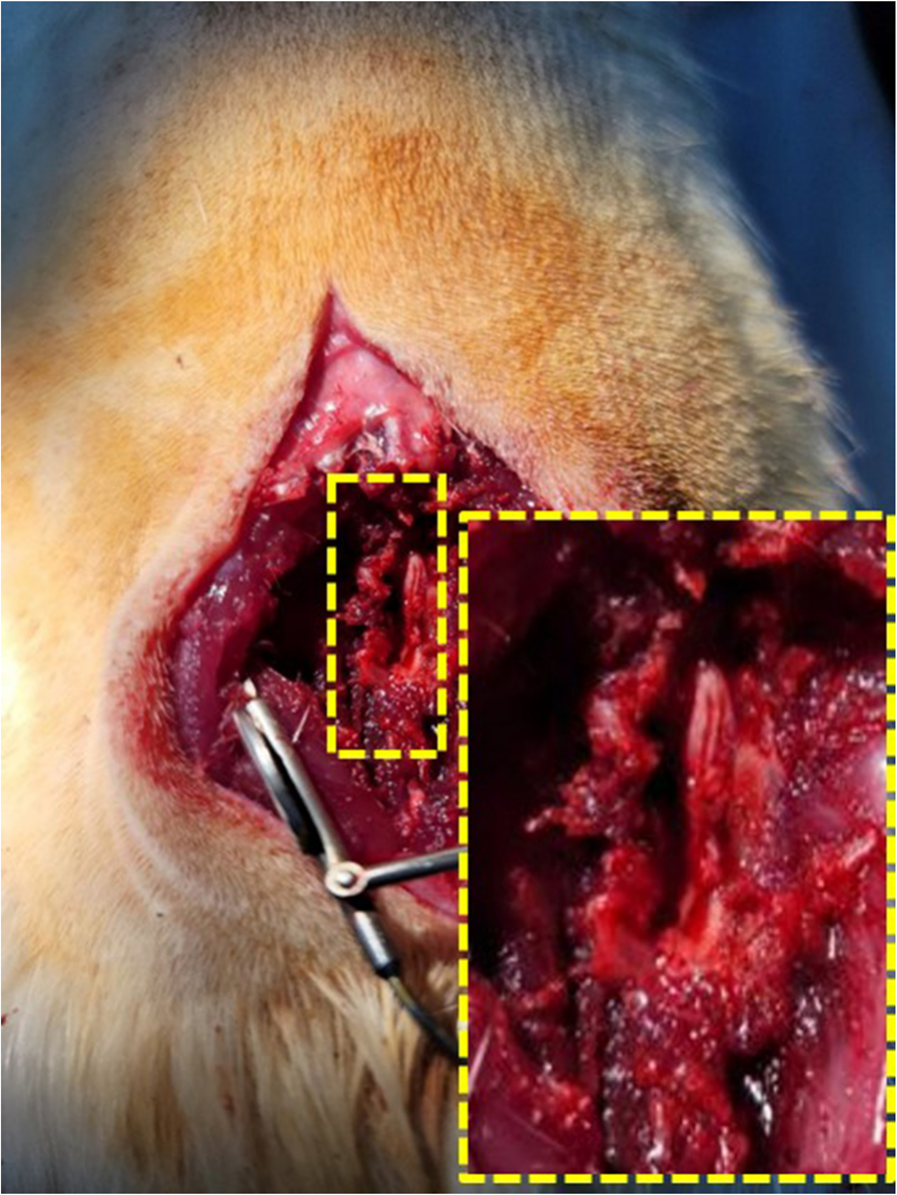
Photograph of laminectomy. S-D rats were anesthetized and subjected to laminectomy at the L4–6 site

All animals were intraperitoneally anaesthetised with 40 mg/kg ketamine hydrochloride (Ketamine 50®; YUHAN, Korea) and 5 mg/kg Rompun injection (Rompun®; BAYER, Korea). After shave the lower back, the rats were sterilised at the surgical site with povidone, and covered with sterile surgical drapes. To expose the bony posterior elements from L4 to L6, a longitudinal midline skin incision was performed, and the lumbosacral fascia was incised and paraspinous muscles were dissected subperiosteally. Then the L4–6 laminectomy and ligamentum flavum were performed, and epidural fat tissue was removed (Fig. 1). The Second and third surgeries were eliminated epidural fibrosis and adhesion, and removed bony growth. The muscle and skin at surgical site was sutured using 4–0 black silk (Aileen, Republic of Korea). During the surgery, all animal’s body temperature was maintained using a warm pad. At the end of the experimental period, all animals were euthanized using CO_2_ gas. The entire laminectomy site was excised according to the spinal cord levels, step by step under a dissecting microscope (Nikon, SMZ800N, Japan). After the removal of the surgical site, L4–5 samples were fixed in a 10% formalin solution and L6 sample (only spinal cord) was stored at − 80 °C in a cryotube for protein extraction.

### Measurement of dural thickness

The fixed specimens were decalcification and dehydration, and preparation of paraffin blocks. Four-μm-thick sections were obtained in the transverse plane and stained with hematoxylin & eosin. Slides were evaluated in a blinded manner by a histologist who analysed dural thickness. Quantitative morphometric analysis was performed on those sections obtained using the Nikon NIS Elements Imaging Software Version 3.22. Dural thickness was measured at 3 points as described by Cemil et al. [18]: the first sample was harvested from the midpoint of the laminectomy defect, the second sample was obtained 2 mm from the right side of the first sample, and the third sample was obtained 2 mm from the left side of the first sample. Mean dural thickness was considered for statistical evaluation.

### Western blotting

For protein extraction, the spinal cord of L6 was placed in RIPA buffer (CBR0002; LPS solution, KOREA) with cOmplete™ EDTA-free protease inhibitor cocktail tablets (0469332001; Sigma-Aldrich, Germany) and shaken in a tissue lyser (TissueLyser II, QIAGEN, Germany) at the rate of 30 times/min for 3 min. The protein content was quantified using BSA, and the extracted and quantified proteins were diluted to 20 μg/μL for western blotting. Then the proteins were loaded onto 10% sulphate-polyacrylamide gels for electrophoresis (#456–1034; Bio-Rad Laboratories, Inc., USA). After transferred to nitrocellulose membranes and blocking, the proteins were incubated with the following primary antibodies overnight at 4 °C: total-ERK1/2 (1:1000; #4348, Cell Signaling Technology, Inc., USA), total-p38 (1:1000; #9212, Cell Signaling Technology, Inc. USA), total-JNK (1:1000; #9258, Cell Signaling Technology, Inc. USA), phosphorylated ERK1/2 (1:2000; #4370, Cell Signaling Technology, Inc. USA), phosphorylated p38 (1:1000; #4511, Cell Signaling Technology, Inc. USA), phosphorylated JNK (1:1000; #9251, Cell Signaling Technology, Inc. USA) and β-actin (1:1000; #5125, Cell Signaling Technology, Inc. USA). After washe three times with TBST, the proteins were incubated with the secondary anti-rabbit IgG HRP-linked antibody (1:5000; #7074, Cell Signaling Technology, Inc. USA) at room temperature for 2 h, and then ECL (iNtRON Biotechnology, Inc., Korea) of the solution was determined.

### Immunohistochemical analysis

Formalin-fixed tissues were decalcified by the 10% nitric acid solution. After deminerazation, the samples were embedded in paraffin, and cut into 4-μm-thicknesses. The prepared slides were dehydrated in xylene and graded ethanol, immersed in citrate buffer and boiled for 10 min using an electronic rage. Immunostaining was performed following the ABC kit manual. After blocking, the slides were incubated with the following primary antibodies at 4 °C overnight: phosphorylated ERK1/2 (1:400; #4370, Cell Signaling Technology, Inc. USA), phosphorylated p38 (1:1600; #4511, Cell Signaling Technology, Inc. USA) and phosphorylated JNK (1:100; #9251, Cell Signaling Technology, Inc. USA). Subsequently, the slides were incubated with a secondary antibody at room temperature for 1 h. All slides were developed using the ImmPACT™ NonaRED™ peroxidase substrate (Sk-4805; Vector Laboratories Inc., USA), mounted and observed under a microscope. To quantify the IHC stain, p-ERK, p-p38 and p-JNK contents were calculated by applying the selected threshold analysis in the positive stain at spine dorsal horns via open-source ImageJ. The positive pixel areas were divided by spine dorsal horn available for the positive stain ratio (positive stain ratio = IHC positive stained area/ the spine dorsal horn area × 100%).

### Statistical analysis

All data were indicated as mean value and standard deviation. Statistical analysis considering a value of *P* < 0.05 was conducted through the single factor analysis of variance (ANOVA) via SPSS v.12.0 software (Chicago, IL, USA).

## Results

### Epidural fibrosis following repeated laminectomy

**Fig. 2:**
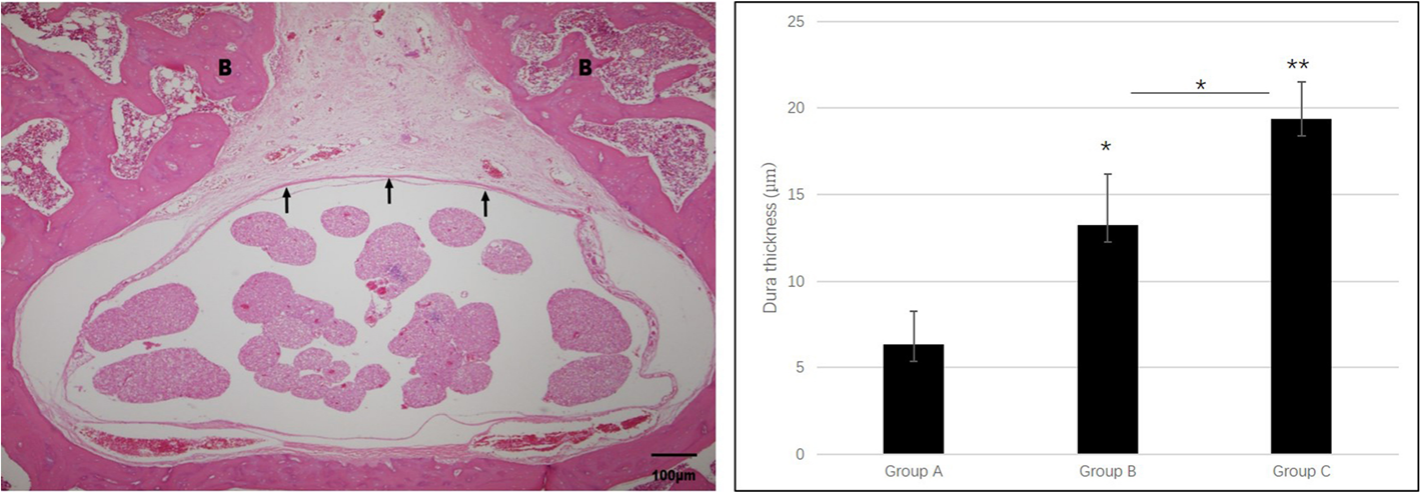
Dural thickness following repeated spinal surgeries (black arrow: dura mater, B: bone. * *p* < 0.05, ** *p* < 0.01)

Epidural fibrosis was indirectly analysed by measuring the dural thickness. The thickness was 6.363 ± 1.911 μm in group A, 13.238 ± 2.123 μm in group B and 19.4 ± 2.115 μm in group C, respectively (Fig. 2). The thickness in group A was significantly lower than that in groups B (*p* < 0.05) and C (*p* < 0.01), and the thickness in group B was significantly lower than that in group C (*p* < 0.05). Therefore, repetitive laminectomy seemingly increases the dural thickness. In other words, repetitive spinal surgerise alone increases epidural fibrosis.

### ERK1/2 protein expression in the spinal cord following repeated laminectomy

In the western blotting, the total-ERK expression was similar across three groups, but compared with group A, group B showed a 1.04-fold increase and group C showed a 0.89-fold increase in expression.

**Fig. 3:**
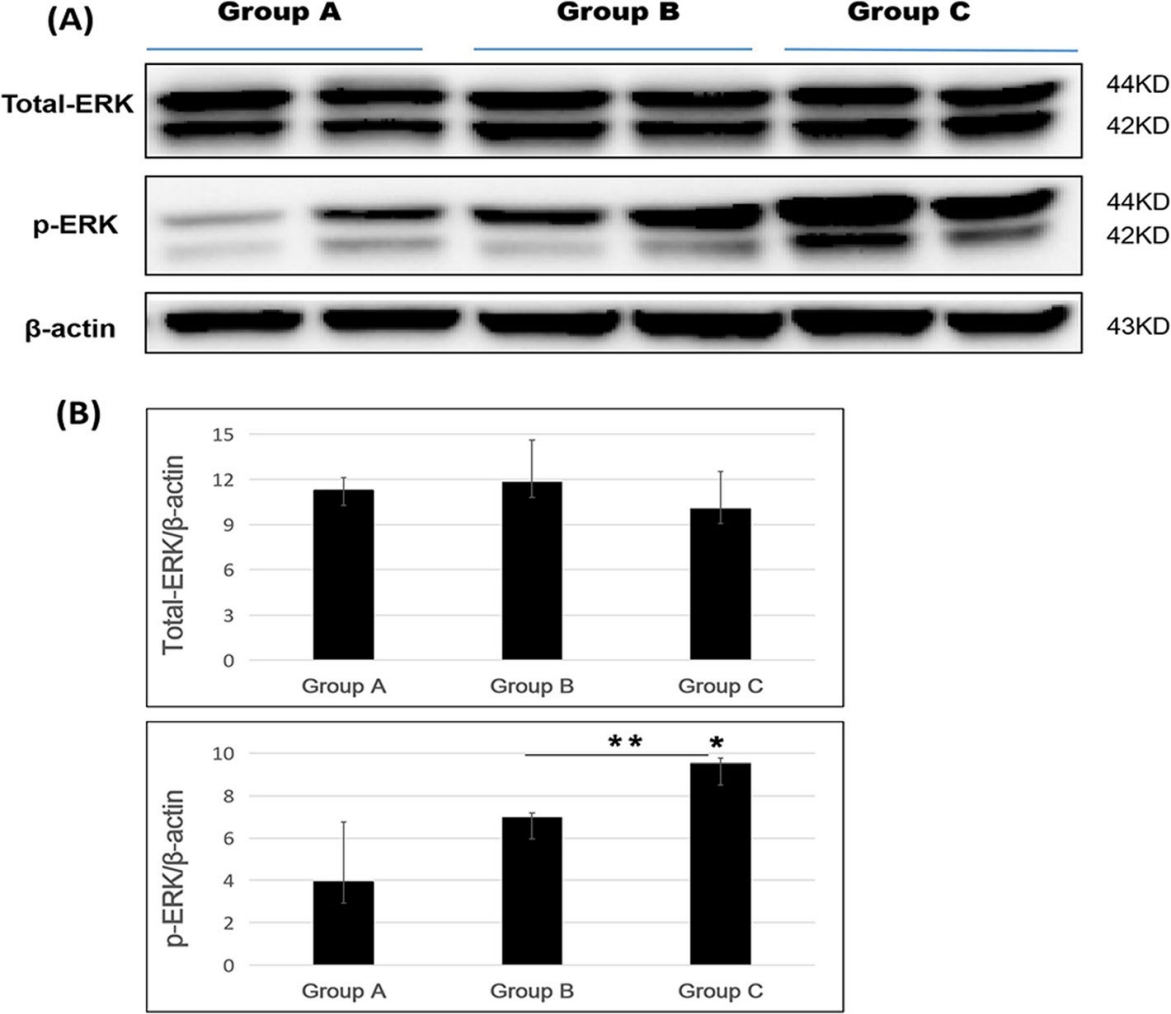
Total-ERK and p-ERK proteins expression following repeated spinal surgeries. **a** Western blotting, and **b** density measurement (* *p* < 0.05, ** *p* < 0.01)

Phosphorylated ERK expression was the lowest in group A; group B showed 1.77-fold increased expression compared with group A, and group C showed the highest expression, with a 2.42-fold increased expression compared with group A (Fig. 3a and b). In addition, phosphorylated ERK expression was significantly different between groups A and C (*p* < 0.05) and between groups B and group C (*p* < 0.01).

Immunohistochemical staining revealed that phosphorylated ERK1/2 was expressed in the spinal dorsal horn in group A with primary surgery, in group B with secondary surgery and in group C with tertiary surgery. Moreover, phosphorylated ERK1/2 expression gradually increased with the number of surgeries, and expression in group B and C was significantly increased as compared to expression in group A (group A: 9.18±1.155 vs. group B: 46.02±11.937, *p* < 0.05; group A: 9.18±1.155 vs. group C: 74.01±6.519, *p* < 0.01; group B vs. group C, *p* < 0.05) (Fig. 6a and b).

It is reported that ERK plays an important role in chronic neuropathic pain. In various pain models, ERK is specifically activated in the superficial dorsal horn neurons [14]. In this study, our results indicate that phosphorylated ERK1/2, which is considered to be involved in causing neuropathic pain, is overexpressed following repeated spinal surgery in the spinal dorsal.

### p38 protein expression in the spinal cord following repeated laminectomy

In the western blotting results, total-p38 expression showed a decreasing trend with the number of spinal surgeries. Group A showed the highest expression, while groups B and C showed 0.81-fold and 0.57-fold decreases compared with group A; expression in groups A and C was significantly different (*p* < 0.05).

**Fig. 4:**
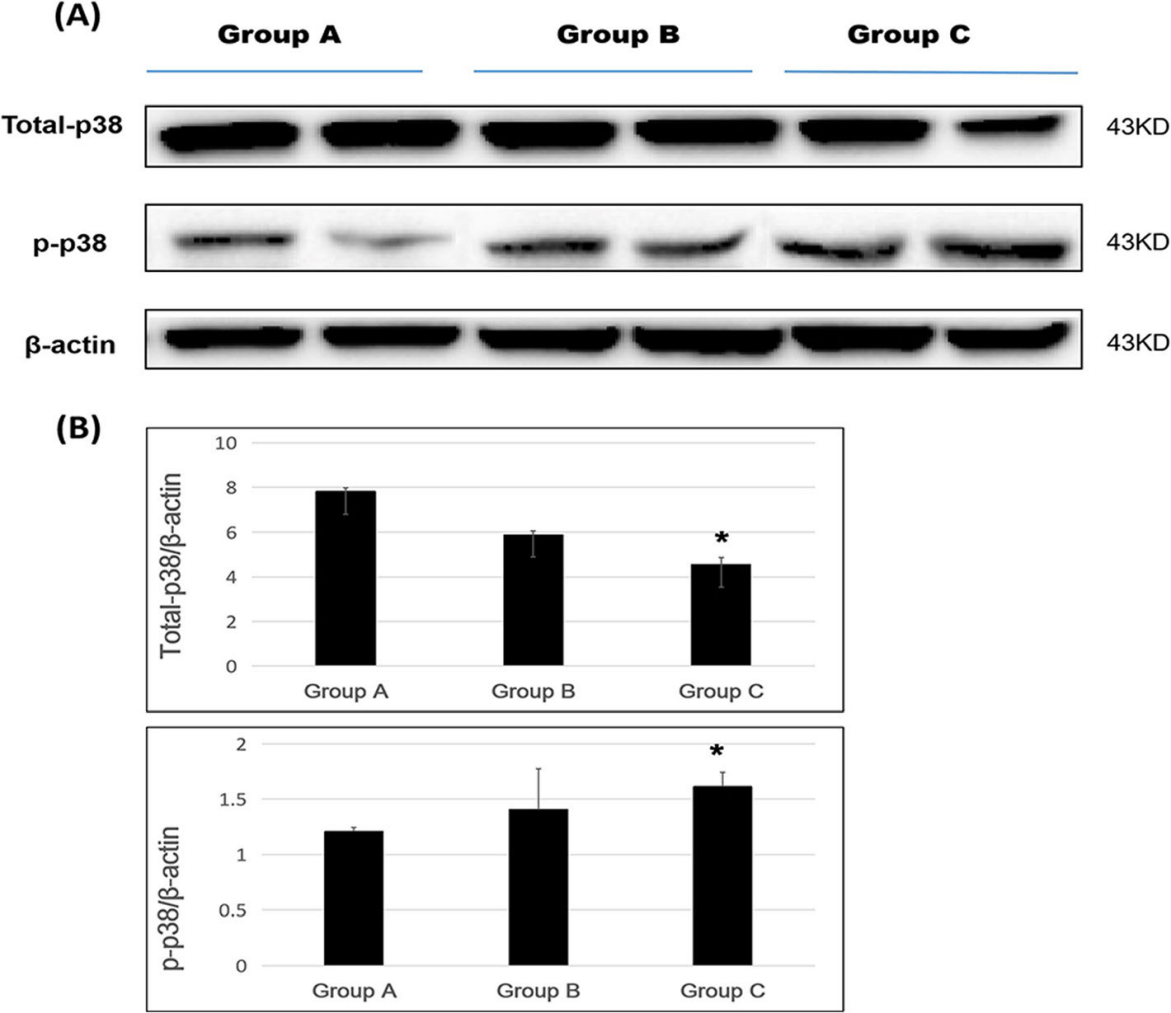
Total-p38 and p-p38 protein expression following repeated spinal surgeries. **a** Western blotting, and **b** density measurement (* *p* < 0.05)

Phosphorylated p38 showed an increasing trend with the number of spinal surgeries. Group A showed the lowest expression, and groups B and C showed a 1.17-fold and 1.33-fold increased expression compared with group A; expression in groups A and C was significantly different (*p* < 0.05) (Fig. 4a and b).

Immunohistochemical staining revealed that phosphorylated p38 was expressed in group A with primary surgery, in group B with secondary surgery and in group C with tertiary surgery. Moreover, phosphorylated p38 expression gradually increased with the number of surgeries, as confirmed by the results of western blotting (Fig. 6a). Moreover, compared with group A, groups B and C were showed significantly increased expression (group A: 9.40±1.673 vs. group B: 32.61±13.831, *p* < 0.05; group A: 9.40±1.673 vs. group C: 50.05±7.906, *p* < 0.01) (Fig. 6b).

All these results abovementioned show that phosphorylated p38, which is considered to be involved in neuropathic pain, is overexpressed following repeated spinal surgeries.

### JNK protein expression in the spinal cord following repeated laminectomy

Western blotting results revealed that total-JNK expression was the lowest in group A, while groups B and C showed 1.64-fold and 1.24-fold increases in expression compared with group A, and the expression in groups A and group B was significantly different (*p* < 0.05).

**Fig. 5:**
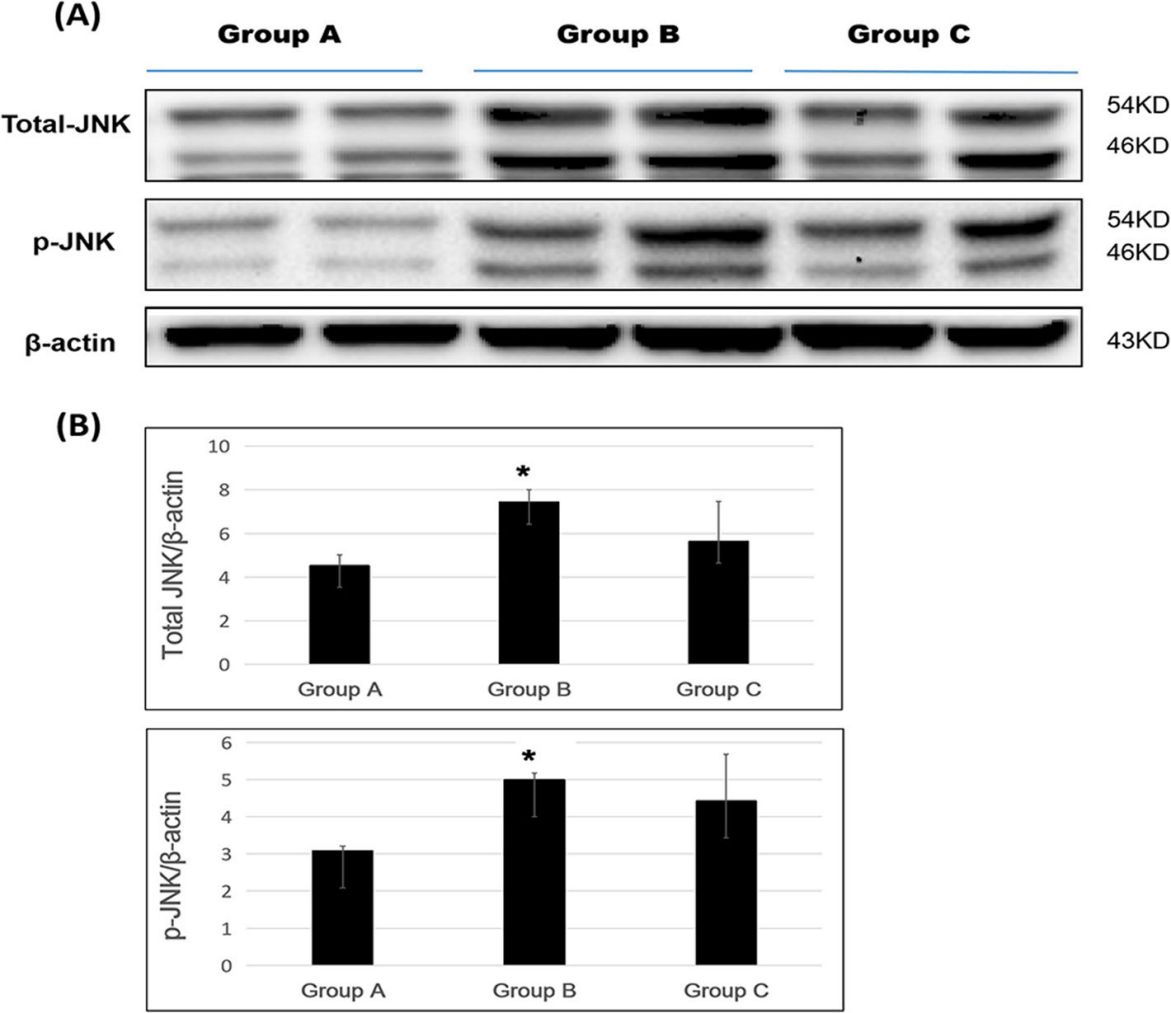
Total-JNK and p-JNK protein expression following repeated spinal surgeries. **a** Western blotting, and **b** density measurement (* *p* < 0.05)

Phosphorylated JNK expression was the lowest in in group A, and compared with group A, groups B and C showed 1.62-fold and 1.43-fold increased expression; expression in groups A and B was significantly different (*p* < 0.05) (Fig. 5a and b).

**Fig. 6:**
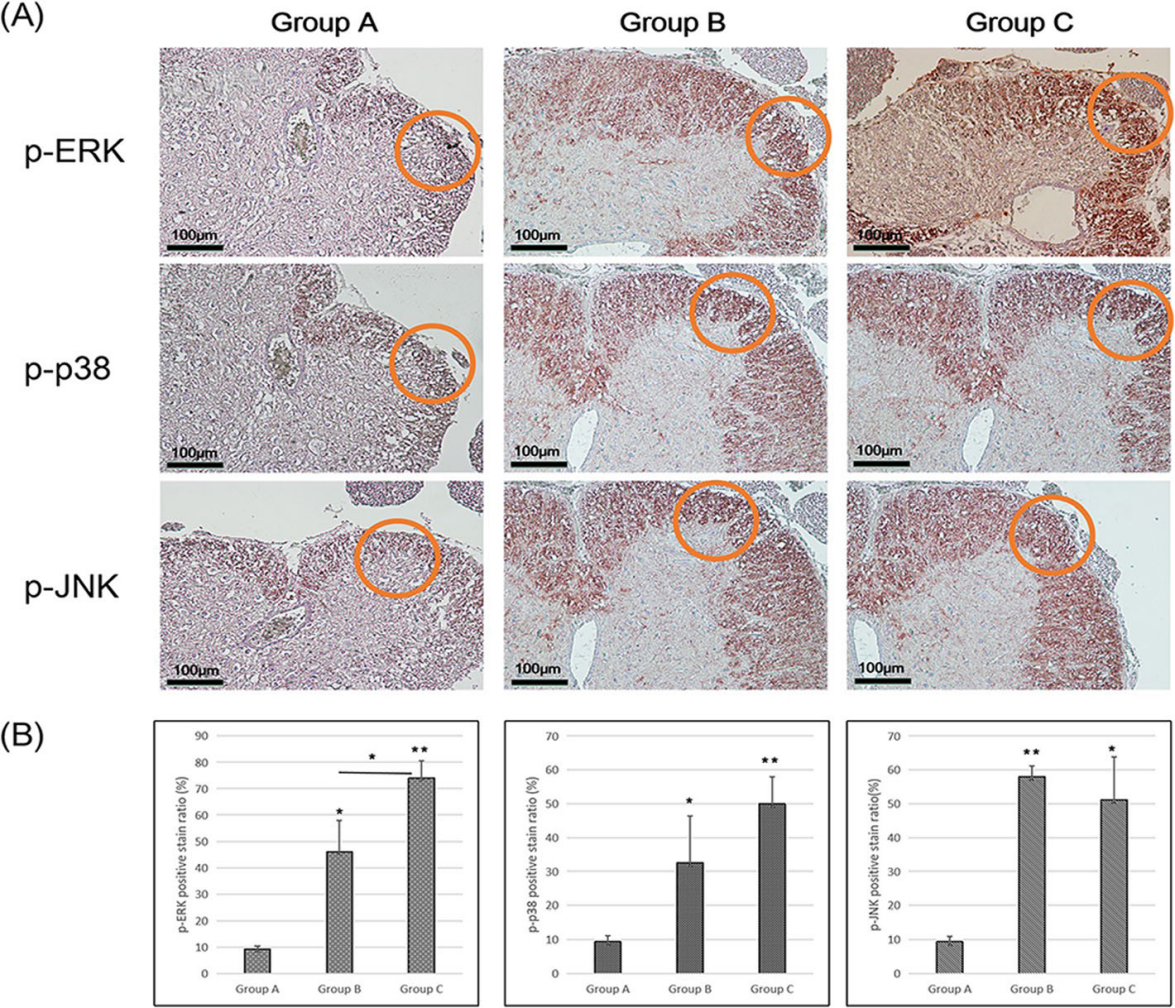
Immunohistochemical analysis of phosphorylated MAPK markers in the spinal dorsal horn. **a** images of immunohistochemical analysis of p-ERK, p-p38 and p-JNK and **b** quantitative assessment of the positive stain ratio (%) (* *p* < 0.05, ** *p* < 0.01; circle: spinal dorsal horn)

Immunohistochemical staining revealed that phosphorylated JNK expression was the lowest in group A. Compared with group A, groups B and C showed increased expression (group A: 9.41±1.581 vs. group B: 58.02±3.082, *p* < 0.01; group A: 9.41±1.581 vs. group C: 51.05±12.558, *p* < 0.05). However, phosphorylated JNK expression in the spinal dorsal horn was higher in group B than in group C, as confirmed by the results of western blotting (Fig. 6a and b).

## Discussion

As shown in the morphological and molecular analysis, this study demonstrates the relationship between pain markers and epidural fibrosis using a rat spinal surgery epidural fibrosis model. Epidural fibrosis, which is a common complication of lumbar disc surgery, causes repeated radicular pain or back pain due to compression of the exposed dura and nerve roots [19]. Epidural fibrosis is the most common cause of FBSS, one should analyse its contribution to symptoms created by stenosis and disc herniation, where a mild stenosis or a simple bulging would not usually cause any symptoms but the presence of epidural fibrosis only helps to worsen the clinical finding [8]. Even with the evolution of MRI and CT imaging, an improvement of surgical technology and sophisticated surgical strategies in spinal diseases, epidural fibrosis is still a conundrum. Because, epidural fibrosis may be the cause of recurrent pain in the majority of cases. Recently, some surgeon tries to approach with indirect decompression in a revision surgery to avoid these scar formations and incidental durotomy complications [20].

Post-laminectomy epidural fibrosis is well known. Turkoglu et al. [7] identified the mechanism of action of etanercept after inducing spinal epidural fibrosis in a rat model post laminectomy. Similarly, Alkalay et al. [21] demonstrated that a post-laminectomy epidural fibrosis model could be used to prevent epidural fibrosis of bioplastic materials. In addition, Kurt et al. [22] used a post-laminectomy epidural fibrosis model to compare the effects of waxed paper and Gore-Tex on the prevention of post-laminectomy epidural fibrosis. Therefore, in the present study, we constructed a rat model of post-laminectomy epidural fibrosis.

In animal models of neuropathic pain caused by peripheral nerve injury, neuropathic pain was not completely manifested at an early stage (i.e. 0–3 days post-lesion), but it was well developed at a later stage (i.e. 7–21 days post-lesion) [21]. In addition, in a partial sciatic nerve ligation model, mechanical allodynia was well established in the affected hind paw at 3 week post-lesion [10, 23]. Therefore, in this study, the reoperation interval was set as 3 weeks. Furthermore, in our experiments, the spinal surgery was repeated once, twice or thrice, and it was confirmed that dural thickness increased with as the number of repeated surgeries. These findings suggest that repeated spinal surgeries may increase epidural fibrosis. Therefore, epidural fibrosis increase with repetitive spinal surgery and may be the cause of recurrent pain.

Increasing studies on MAPKs have explored their roles in the generation of chronic central neuropathic pain due to the spinal cord injury [24–26]. In addition, MAPKs such as ERK and p38 was reported to contribute to dorsal horn hyperexcitability in a peripheral neuropathic pain model [27–30]. Thus, in the present study, we evaluated MAPK expression in the spinal cord following repetitive surgeries.

The ERK/MAPK pathway plays an important role in cell proliferation and differentiation. Additionally, the activation of ERK/MAPK signalling contributes to the pain response of the dorsal horn and dorsal root ganglia following inflammation and/or nerve injury [11]. Kondo et al. [31] suggest the important role of ERK signalling in pain perception and the use of phosphorylated ERK as a biomarker for activated cells involved in pain signalling to the somatosensory cortex and nociceptive reflex. Zhuang et al. [30] suggested that ERK acts on neurons, microglia and astrocytes via spinal nerve ligation as well as contributes to mechanical allodynia in a neuropathic pain model. Likewise, in our study, ERK was expressed following repeated spinal surgeries, and the protein expression of phosphorylated ERK gradually increased with the number of repetitions of the surgery, suggesting that phosphorylated ERK contributes to pain development due to epidural fibrosis.

Phosphorylated p38/MAPK induction by nerve injury mainly occurs in the spinal dorsal horn and dorsal root ganglia, which has been extensively studied in terms of the initiation and maintenance of neuropathic pain [10]. Jin et al. [32] used phosphorylated p38 antibody to confirmed p38 activation in the rat spinal cord after spinal never ligation, and suggested that p38 is activated after a spinal nerve ligation in spinal corf microglia and dorsal root ganglion neurons and contributes to the generation of neuropathic pain. Ji et al. [33] reported, phosphorylated p38 levels are low in non-injured spinal cord, spinal nerve ligation induces a substantial increase in phosphorylated p38 levels in the injury side of the spinal cord. In our study, p38 was expressed following repeated spinal surgeries, and the protein expression of phosphorylated p38 gradually increased with the number of repetitions of the surgery. These data suggest that the phosphorylated p38 is actively involved in the pain-related processes due to epidural fibrosis.

Regarding neuropathic pain, the role of JNK is less known than those of ERK and p38, and only a few studies are conducted. Zhuang et al. [17] reported that JNK acts on the sensory nerves and astrocytes to develop and maintain neuropathic pain. However, Crown et al. [24] reported that increases in expression of activated forms of ERK1/2 and p38 but not JNK are correlated with the expression of at-level mechanical allodynia following a spinal cord injury. In our study, JNK was expressed in the spinal dorsal horn following repeated spinal surgeries, but its expression level did not gradually increase with the number of repeated surgeries. Moreover, protein expression level of phosphorylated JNK did not increase with the number of repeated surgeries. Therefore, we believe that JNK may not contribute to pain development due to epidural fibrosis.

In addition, the phosphorylated ERK 1/2 and p38 are reportedly upregulated in similar regions of the spinal cord in injured rats, which induced mechanical allodynia [24]. Choi et al. [34] have demonstrated that the analgesic effect of acupuncture may be partly mediated by inhibiting ROS-induced ERK and p38 activation after a spinal cord injury in rats. These findings suggest that activated ERK1/2 and p38 regulate changes in nociceptive reactivity in peripheral nerve injury models [35–37]. In our experiments, phosphorylated ERK and p38 were expressed in the spinal dorsal horn. Furthermore, in the present study, the expression of phosphorylated ERK and p38 was upregulated as the number of repeated surgeries increased, suggesting that p38 and ERK contribute to pain development due to epidural fibrosis.

Repetitive spinal surgeries are stimulated by the spinal dorsal horn, resulting in increased phosphorylated ERK1/2 and p38 expression. Thus, neuropathic pain is likely induced by epidural fibrosis, and phosphorylated ERK1/2 and p38 are the potential pain-related factors.

## Conclusions

This study was the first to analyse the association between pain markers and epidural fibrosis caused by repeated spinal surgeries in rats. Results indicates that repeated spinal surgeries increase dural thickness, ultimately leading to epidural fibrosis. Moreover, repeated spinal surgeries increased expressions of pain markers such as ERK and p38, indicating that pain increased with the repeated surgeries. However, the DRG was not focussed upon in this study because repeated surgeries does not define the anatomical region of the DRG. Thus, further animal study is needed in the future to evaluate DRG function and test pain behaviour.

## Supplementary Information

**Additional file 1.**

**Additional file 2.**

**Additional file 3.**

## Data Availability

Authors declares that data and materials in the study are available from the corresponding author Y.Y. Kim on reasonable request.

## Abbreviations

DRG: Dorsal root ganglion
FBSS: Failed back surgery syndrome
MAPKs: Mitogen-activated protein kinases
ERK1/2: Extracellular signal-regulated kinases 1/2
JNK: c-Jun N-terminal

## References

[1] Farrokhi MR, Vasei M, Fareghbal S, Farrokhi N (2011). The effect of methylene blue on peridural fibrosis formation after laminectomy in rats: an experimental novel study. Spine J.

[2] Fiume D, Sherkat S, Callovini GM, Parziale G, Gazzeri G (1995). Treatment of the failed back surgery syndrome due to lumbo-sacral epidural fibrosis. Acta Neurochir Suppl.

[3] Richter HP, Kast E, Tomczak R, Besenfelder W, Gaus W (2001). Results of applying ADCON-L gel after lumbar discectomy: the German ADCON-L study. J Neurosurg.

[4] Burton CV (1978). Lumbosacral arachnoiditis. Spine (Phila Pa 1976).

[5] Karanci T, Kelten B, Karaoglan A, Cinar N, Midi A, Antar V (2017). Effects of 4% icodextrin on experimental spinal epidural fibrosis. Turk Neurosurg.

[6] Hayek SM, Helm S, Benyamin RM, Singh V, Bryce DA, Smith HS (2009). Effectiveness of spinal endoscopic adhesiolysis in post lumbar surgery syndrome: a systematic review. Pain Physician.

[7] Turkoglu E, Tuncer C, Dinc C, Serbes G, Oktay M, Sekerci Z (2014). The effect of etanercept on spinal epidural fibrosis in a postlaminectomy rat model. Turk Neurosurg..

[8] Ozturk Y, Bozkurt I, Yaman ME, Guvenc Y, Tolunay T, Bayram P (2018). Histopathologic analysis of Tamoxifen on epidural fibrosis. World Neurosurg.

[9] Tuncer C, Subasi C, Dinc C, Turkogly E, Er U (2019). The preventative effect of α-Tocopherol on spinal epidural fibrosis after laminectomy in a rat model. Turk Neurosurg..

[10] Ma W, Quirion R (2005). The ERK/MAPK pathway, as a target for the treatment of neuropathic pain. Expert Opin Ther Targets.

[11] Obata K, Noguchi K (2004). MAPK activation in nociceptive neurons and pain hypersensitivity. Life Sci.

[12] Cargnello M, Roux PP (2011). Activation and function of the MAPKs and their substrates, the MAPK-activated protein kinases. Microbiol Mol Biol Rev.

[13] Lewis TS, Shapiro PS, Ahn NG (1998). Signal transduction through MAP kinase cascades. Adv Cancer Res.

[14] Peng G, Han M, Du Y, Lin A, Yu L, Zhang Y (2009). SIP30 is regulated by ERK in peripheral nerve injury-induced neuropathic pain. J Biol Chem.

[15] Ji RR, Strichartz G (2004). Cell signaling and the genesis of neuropathic pain. Sci STKE.

[16] Ji RR, Woolf CJ (2001). Neuronal plasticity and signal transduction in nociceptive neurons: implications for the initiation and maintenance of pathological pain. Neurobiol Dis.

[17] Zhuang ZY, Wen YR, Zhang DR, Borsello T, Bonny C, Strichartz GR (2006). A peptide c-Jun N-terminal kinase (JNK) inhibitor blocks mechanical allodynia after spinal nerve ligation: respective roles of JNK activation in primary sensory neurons and spinal astrocytes for neuropathic pain development and maintenance. J Neurosci.

[18] Cemil B, Tun K, Kaptanoglu E, Kaymaz F, Cevirgen B, Comert A (2009). Use of pimecrolimus to prevent epidural fibrosis in a postlaminectomy rat model. J Neurosurg Spine.

[19] Ozdemir O, Calisaneller T, Sonmez E, Kiyici H, Caner H, Altinors N (2010). Topical use of colchicine to prevent spinal epidural fibrosis in rats. Neurol Res.

[20] Nakashima H, Kanemura T, Satake K, Ito K, Ishikawa Y, Ouchida J (2020). Indirect decompression using lateral lumbar Interbody fusion for restenosis after an initial decompression surgery. ASJ..

[21] Alkalay RN, Kim DH, Urry DW, Xu J, Parker TM, Glazer PA (2003). Prevention of postlaminectomy epidural fibrosis using bioelastic materials. Spine (Phila Pa 1976).

[22] Kurt G, Celik B, Cemil B, Doğulu F, Baykaner MK, Ceviker N (2009). A comparison of the effectiveness of waxed paper and Gore-Tex on the minimally invasive epidural fibrosis model. J Spinal Disord Tech.

[23] Seltzer Z, Dubner R, Shir Y (1990). A novel behavioral model of neuropathic pain disorders produced in rats b partial sciatic nerve injury. Pain..

[24] Crown ED, Ye Z, Johnson KM, Xu GY, McAdoo DJ, Hulsebosch CE (2006). Increases in the activated forms of ERK 1/2, p38 MAPK, and CREB are correlated with the expression of at-level mechanical allodynia following spinal cord injury. Exp Neurol.

[25] Crown ED, Ye Z, Johnson KM, Xu GY, McAdoo DJ, Westlund KN (2005). Upregulation of the phosphorylated form of CREB in spinothalamic tract cells following spinal cord injury: relation to central neuropathic pain. Neurosci Lett.

[26] Yu CG, Yezierski RP (2005). Activation of the ERK1/2 signaling cascade by excitotoxic spinal cord injury. Brain Res Mol Brain Res.

[27] Ji RR (2004). Peripheral and central mechanisms of inflammatory pain, with emphasis on MAP kinases. Curr Drug Targets Inflamm Allergy.

[28] Ji RR, Kohno T, Moore KA, Woolf CJ (2003). Central sensitization and LTP: do pain and memory share similar mechanisms?. Trends Neurosci.

[29] Zhao P, Waxman SG, Hains BC (2007). Extracellular signal-regulated kinase-regulated microglia-neuron signaling by prostaglandin E2 contributes to pain after spinal cord injury. J Neurosci.

[30] Zhuang ZY, Gerner P, Woolf C, Ji RR (2005). ERK is sequentially activated in neurons, microglia, and astrocytes by spinal nerve ligation and contributes to mechanical allodynia in this neuropathic pain model. Pain..

[31] Kondo M, Shibuta I (2020). Exreacellular signal-regulated kinases (ERK) 1 and 2 as a key molecule in pain research. J Oral Sci.

[32] Jin SX, Zhuang ZY, Woolf CJ, Ji RR (2003). P38 mitogen-activated protein kinase is activated after a spinal nerve ligation in spinal cord microglia and dorsal root ganglion neurons and contributes to the generation of neuropathic pain. J Neurosci.

[33] Ji RR, Suter MR (2007). p38 MAPK, microglial signalling, and neuropathic pain. Mol Pain.

[34] Choi DC, Lee JY, Lim EJ, Baik HH, Oh TH, Yune TY (2012). Inhibition of ROS-induced p38MAPK and ERK activation in microglia by acupuncture relieves neuropathic pain after spinal cord injury in rats. Exp Neurol.

[35] Kim SY, Bae JC, Kim JY, Lee HL, Lee KM, Kim DS (2002). Activation of p38 MAP kinase in the rat dorsal root ganglia and spinal cord following peripheral inflammation and nerve injury. NeuroReport..

[36] Song XS, Xu YB, Cao JL, He JH, Zhang LC, Zeng YM (2005). cAMP response-element binding protein participates in the phosphorylated extracellular signal-regulate kinase mediated neuropathic pain. Sheng Li Xue Bao.

[37] Zhang FE, Cao JL, Zhang LC, Zeng YM (2005). Activation of p38 mitogen-activated protein kinase in spinal cord contributes to chronic constriction injury-induced neuropathic pain. Sheng Li Xue Bao.

