# Supplementary figures and images for: Analysis of pain markers and epidural fibrosis caused by repeated spinal surgery in Sprague–Dawley rats
Source: BMC Musculoskelet Disord. 2021 Jan 5;22:16. doi: 10.1186/s12891-020-03920-z (PMC7786924; doi:10.1186/s12891-020-03920-z)

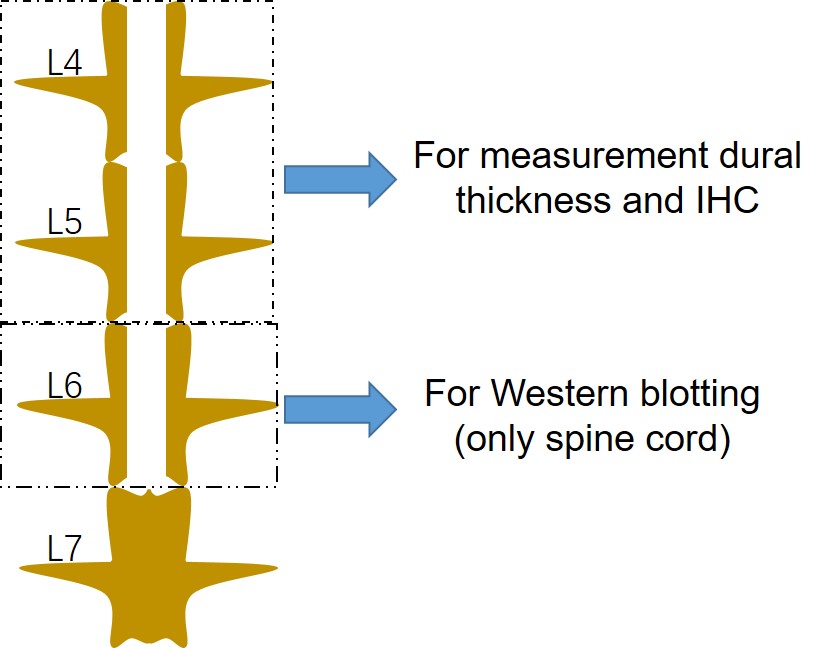

Supplement: Supplementary file 1 — Additional file 1. [file 12891_2020_3920_MOESM1_ESM.jpg]

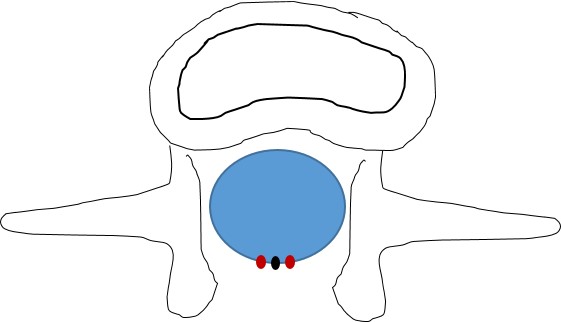

Supplement: Supplementary file 2 — Additional file 2. [file 12891_2020_3920_MOESM2_ESM.jpg]
