# Supplementary material for: Analysis of pain markers and epidural fibrosis caused by repeated spinal surgery in Sprague–Dawley rats
Source: BMC Musculoskelet Disord. 2021 Jan 5;22:16. doi: 10.1186/s12891-020-03920-z (PMC7786924; doi:10.1186/s12891-020-03920-z)
Supplement: Supplementary file 3 — Additional file 3. [file 12891_2020_3920_MOESM3_ESM.pptx]

## Slide 1
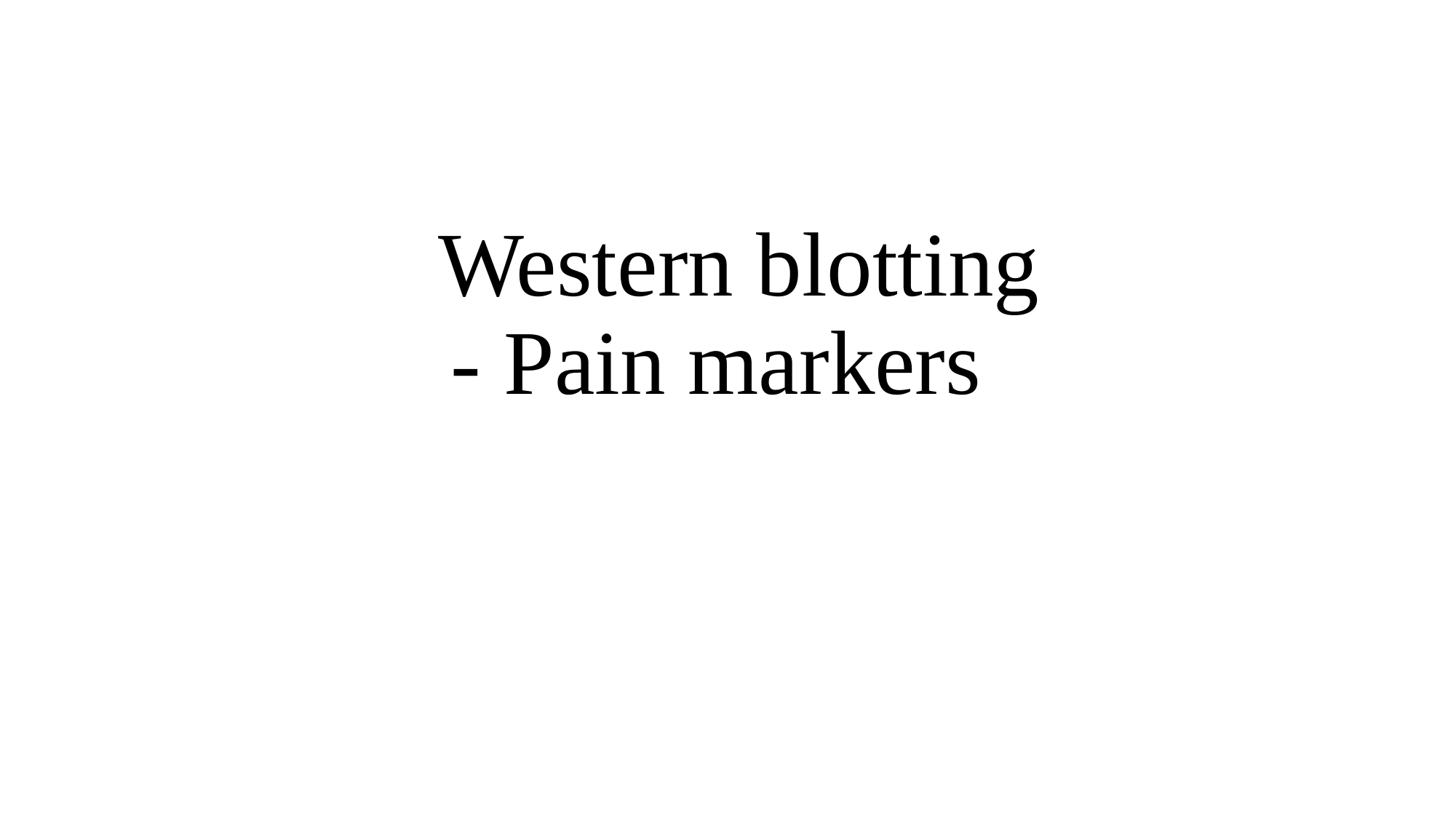

# Western blotting - Pain markers

## Slide 2
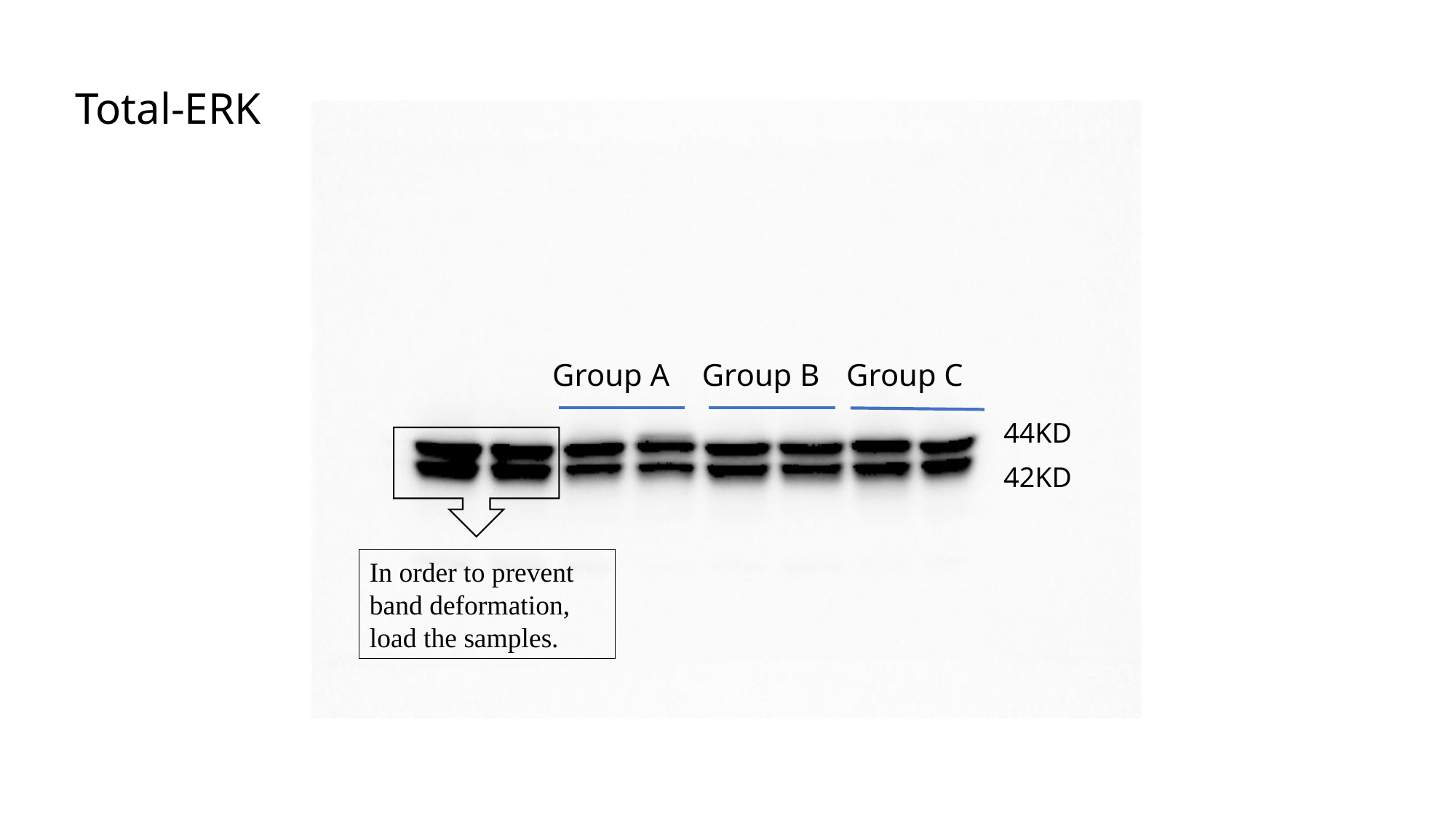

Total-ERK
Group A
Group B
Group C
44KD
42KD
In order to prevent band deformation, load the samples.

## Slide 3
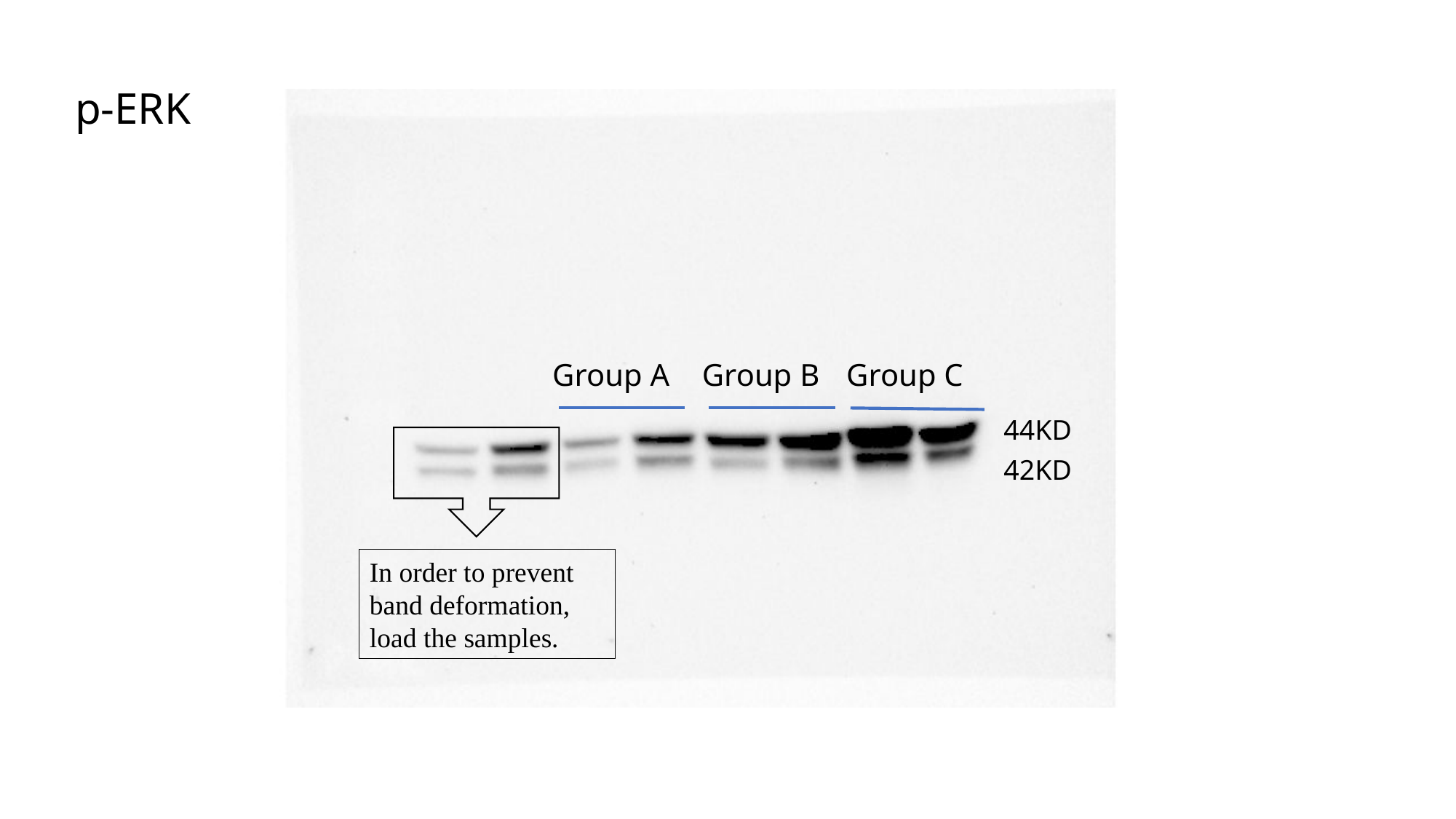

p-ERK
Group A
Group B
Group C
44KD
42KD
In order to prevent band deformation, load the samples.

## Slide 4
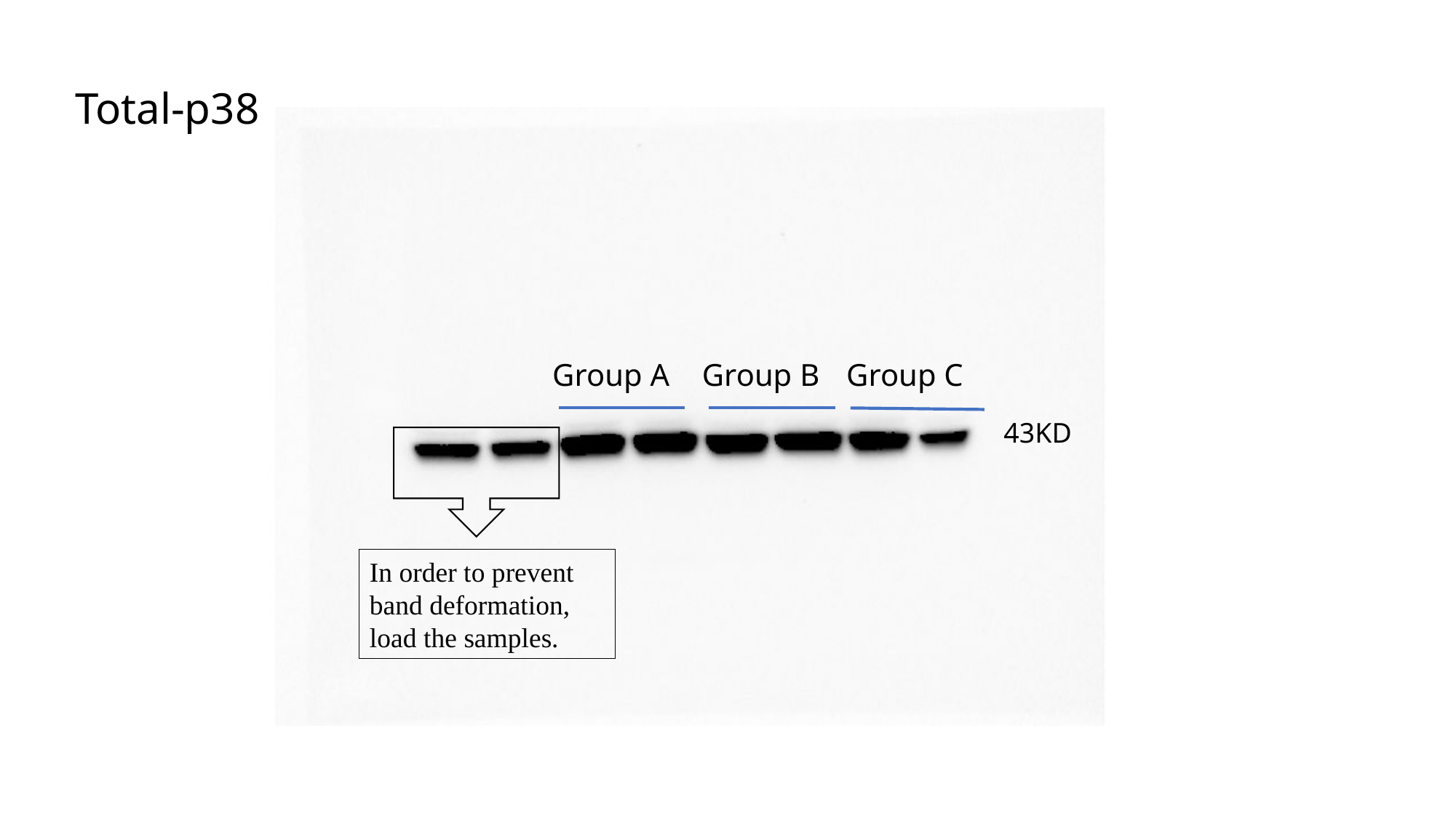

Total-p38
Group A
Group B
Group C
43KD
In order to prevent band deformation, load the samples.

## Slide 5
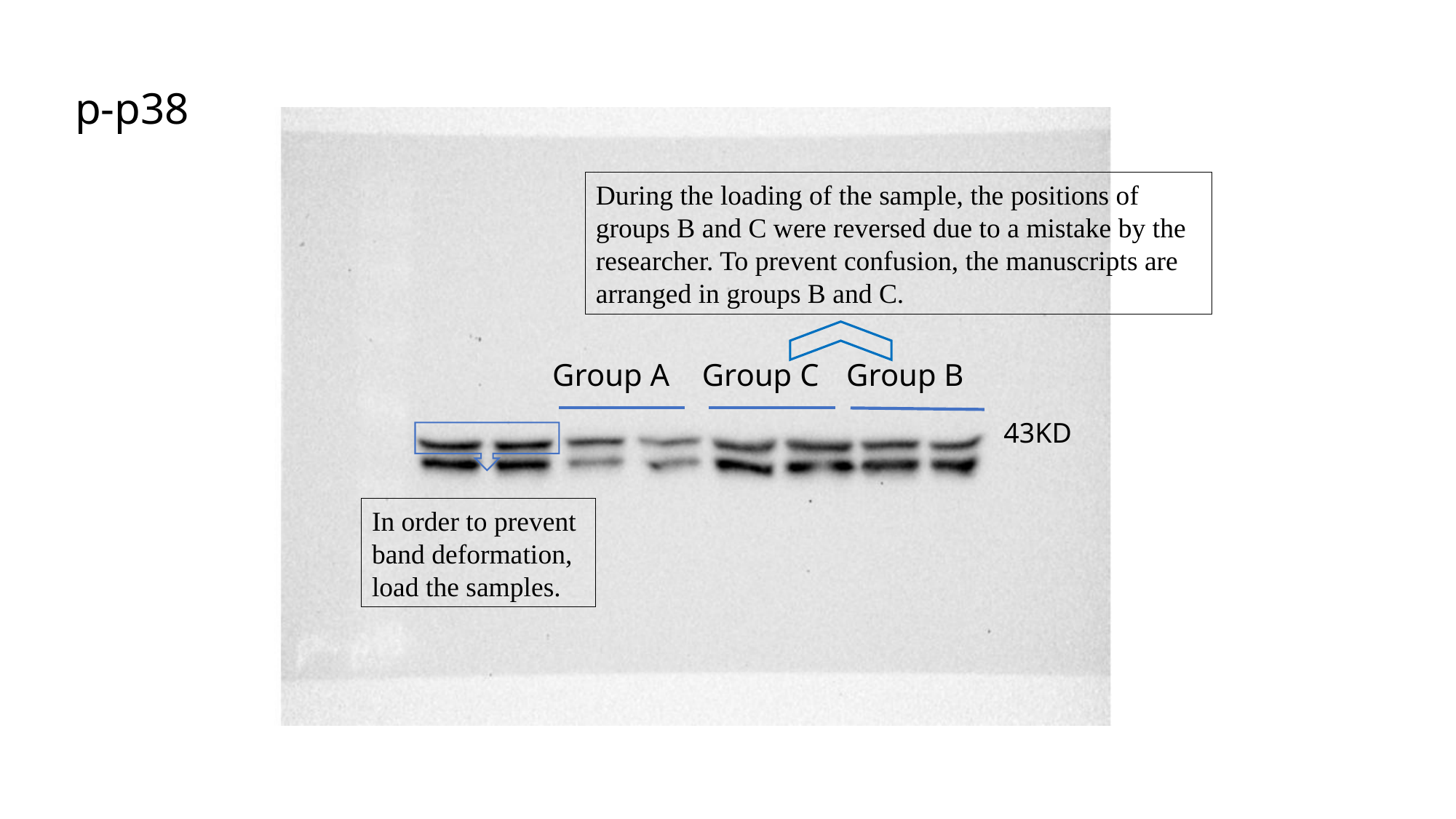

p-p38
During the loading of the sample, the positions of groups B and C were reversed due to a mistake by the researcher. To prevent confusion, the manuscripts are arranged in groups B and C.
Group A
Group C
Group B
43KD
In order to prevent band deformation, load the samples.

## Slide 6
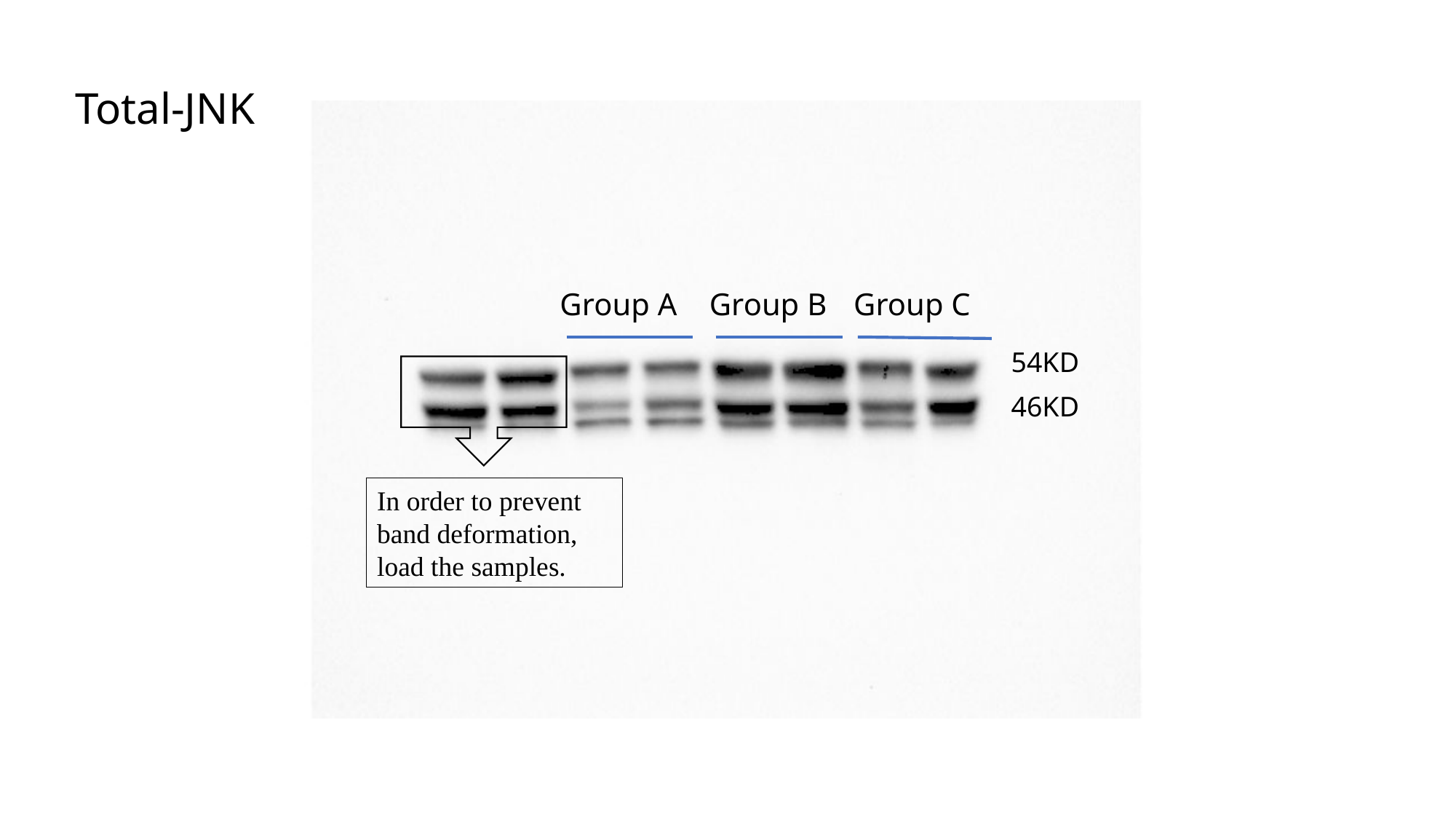

Total-JNK
Group A
Group B
Group C
54KD
46KD
In order to prevent band deformation, load the samples.

## Slide 7
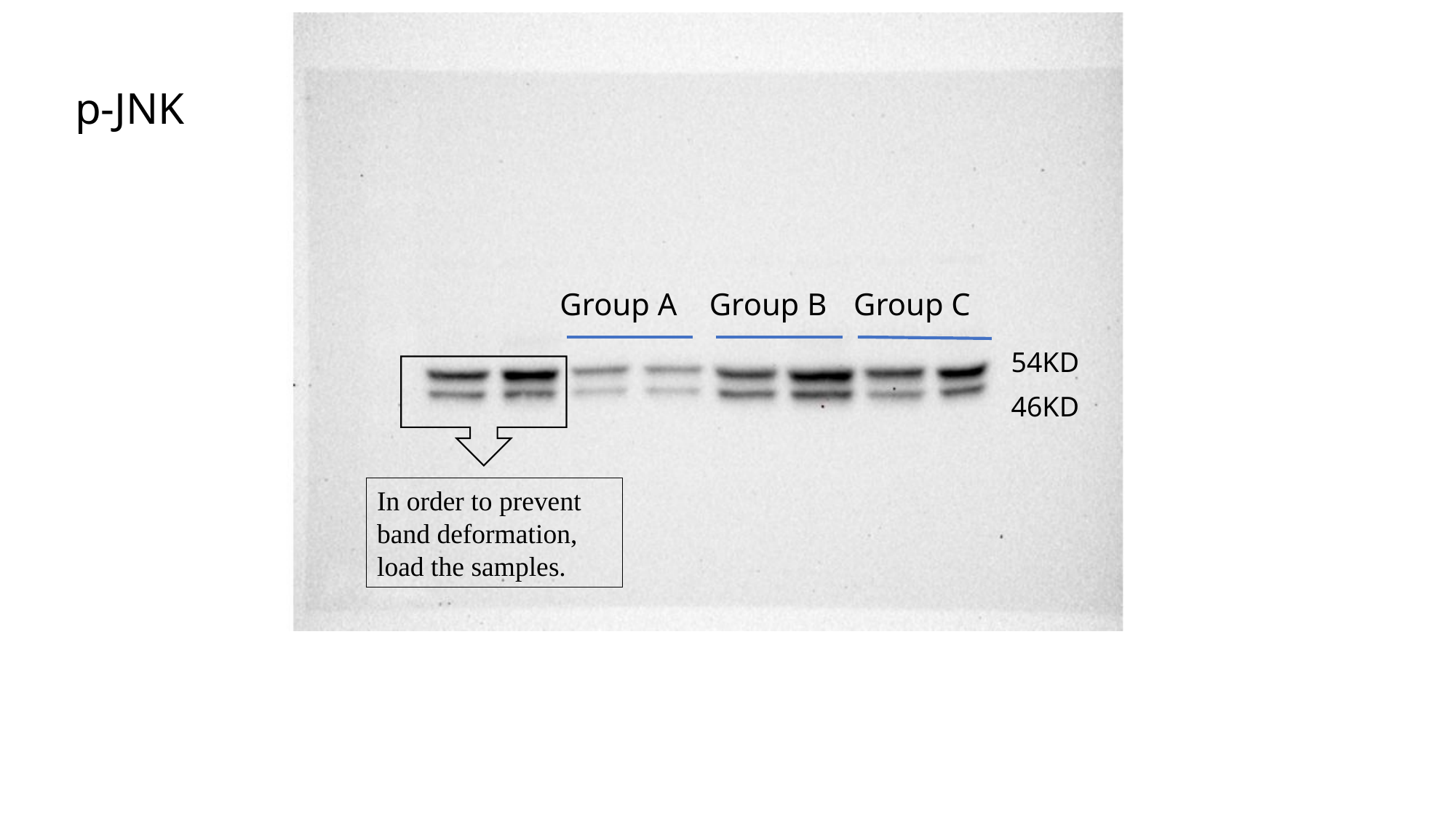

p-JNK
Group A
Group B
Group C
54KD
46KD
In order to prevent band deformation, load the samples.

## Slide 8
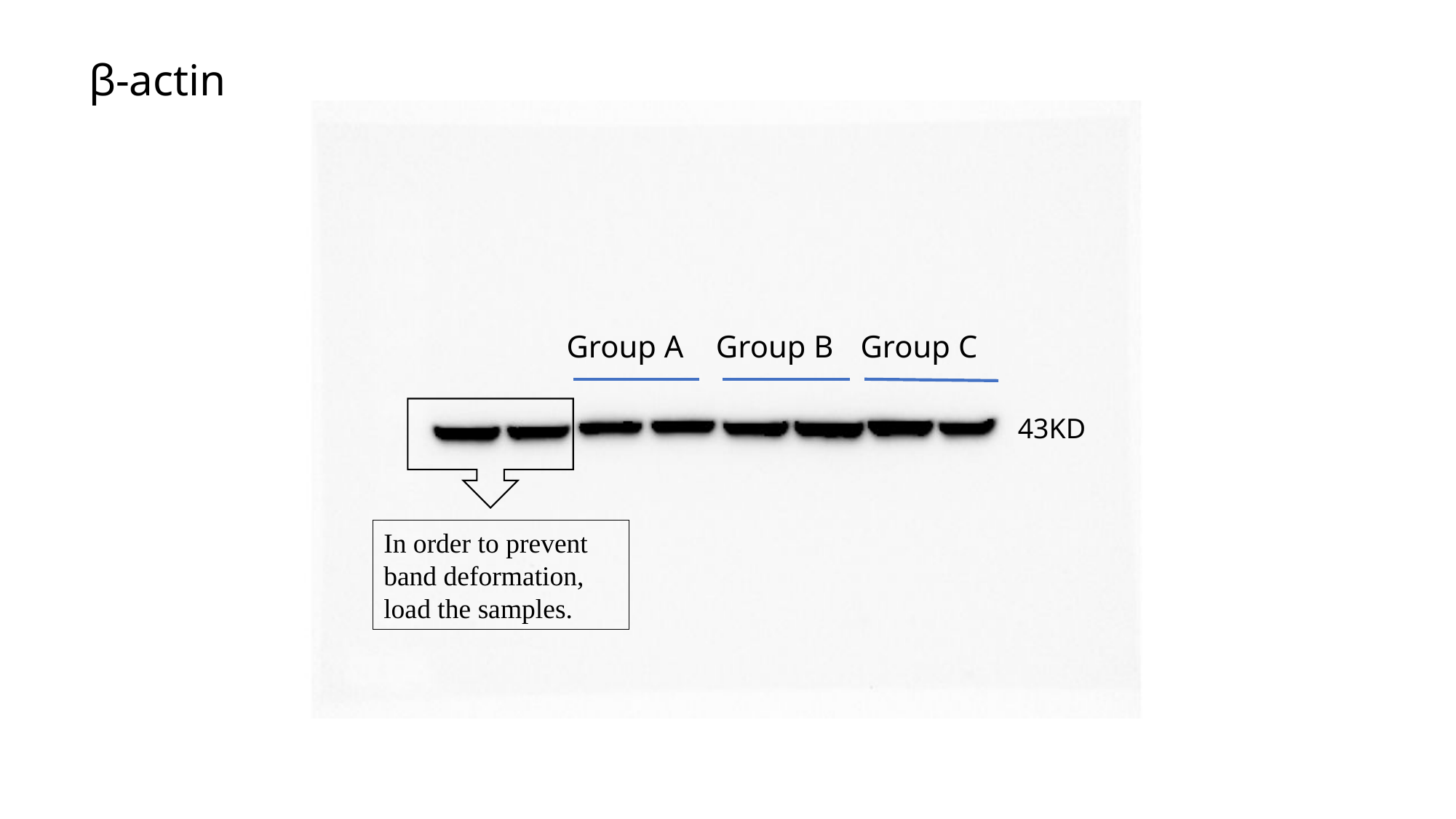

β-actin
Group A
Group B
Group C
43KD
In order to prevent band deformation, load the samples.
